# Hovenia dulcis: a Chinese medicine that plays an essential role in alcohol-associated liver disease

**DOI:** 10.3389/fphar.2024.1337633

**Published:** 2024-04-08

**Authors:** Yi-Xiang He, Meng-Nan Liu, Yang-Yang Wang, Hao Wu, Mei Wei, Jin-Yi Xue, Yuan Zou, Xin Zhou, Hui Chen, Zhi Li

**Affiliations:** ^1^ The Key Laboratory of Integrated Traditional Chinese and Western Medicine for Prevention and Treatment of Digestive System Diseases of Luzhou City, Affiliated Traditional Medicine Hospital of Southwest Medical University, Luzhou, Sichuan, China; ^2^ College of Integrated Chinese and Western Medicine, Southwest Medical University, Luzhou, Sichuan, China; ^3^ Department of Spleen and Stomach Diseases, The Affiliated Traditional Chinese Medicine Hospital of Southwest Medical University, Luzhou, Sichuan, China

**Keywords:** alcohol-associated liver disease, Hovenia dulcis, mechanisms of action, Chinese herbal, treatment of alcohol-associated liver disease

## Abstract

Globally, alcohol-associated liver disease (ALD) has become an increased burden for society. Disulfirams, Benzodiazepines (BZDs), and corticosteroids are commonly used to treat ALD. However, the occurrence of side effects such as hepatotoxicity and dependence, impedes the achievement of desirable and optimal therapeutic efficacy. Therefore, there is an urgent need for more effective and safer treatments. Hovenia dulcis is an herbal medicine promoting alcohol removal clearance, lipid-lowering, anti-inflammatory, and hepatoprotective properties. Hovenia dulcis has a variety of chemical components such as dihydromyricetin, quercetin and beta-sitosterol, which can affect ALD through multiple pathways, including ethanol metabolism, immune response, hepatic fibrosis, oxidative stress, autophagy, lipid metabolism, and intestinal barrier, suggesting its promising role in the treatment of ALD. Thus, this work aims to comprehensively review the chemical composition of Hovenia dulcis and the molecular mechanisms involved in the process of ALD treatment.

## 1 Introduction

Alcohol-associated liver disease (ALD) is strongly associated with chronic excessive alcohol consumption (Liu S. Y. et al., 2021). The World Health Organization (WHO) (Jiaming Chen, 2022) has estimated that approximately 2.3 billion individuals worldwide are currently engaged in alcohol consumption, with a significant proportion found in the United States, Europe, and the Western Pacific. This global trend has significant implications for the progression and outcomes of more than 200 diseases (Liu S. Y. et al., 2021). Moreover, it contributes to increased morbidity, mortality, and financial burden on society, resulting in over 3 million deaths annually (Zhao et al., 2018). The prevalence of ALD in China has significantly risen, making it the second country in terms of high liver disease prevalence after viral hepatitis (Liu et al., 2010). Regional epidemiological studies have indicated that the prevalence of ALD varies from 0.50% to 8.55% in different regions (Tang et al., 2023). Due to the escalating prevalence and severe implications of ALD, timely intervention and treatment of ALD are crucial for improving its prognosis.

*Hovenia dulcis* Thunb. (Rhamnaceae) (Hovenia dulcis) (botanical name approved by http://mpns.kew.org) is a kind of traditional Chinese medicine (TCM) widely used in China, Korea, and Japan, and is also used as ingredients for food supplements and nutraceuticals (Sferrazza et al., 2021). In the Materia Medica Compendium (A.D. 1,578), Hovenia dulcis is listed as a TCM with anti-alcoholic properties (Fangfang Xu and Wang, 2011; Liang and Olsen, 2014). In ancient Chinese medicine, its fruits and pedicels were also used as a febrifuge and administered for parasitic infections, antispasmodic, laxative and diuretic. The seeds were used as a diuretic and were also effective in alcoholism (Patricia et al., 2017) Hovenia dulcis is found in open fields and forests in China at an altitude of less than 2,100 m and is classified as a plant used for both food and medicine (Fangfang Xu and Wang, 2011; Linzhi Kang and Li, 2022). Its chemical components include dihydromyricetin (DHM), quercetin, naringenin, etc., which have been shown to have antioxidant and anti-inflammatory properties and are also considered potential therapeutic agents for alcohol-associated liver disease (Qiu et al., 2019; Meng et al., 2020).

This paper aims to elucidate the fundamental mechanisms underlying ALD and the existing drug treatment strategies, with a specific focus on the impact of Hovenia dulcis‘s chemical constituents on the pathogenesis of ALD through various pathways and targets. It is our aspiration that this knowledge will contribute to future research endeavors in the field of ALD treatment.

## 2 Phytochemistry

**FIGURE 1:**
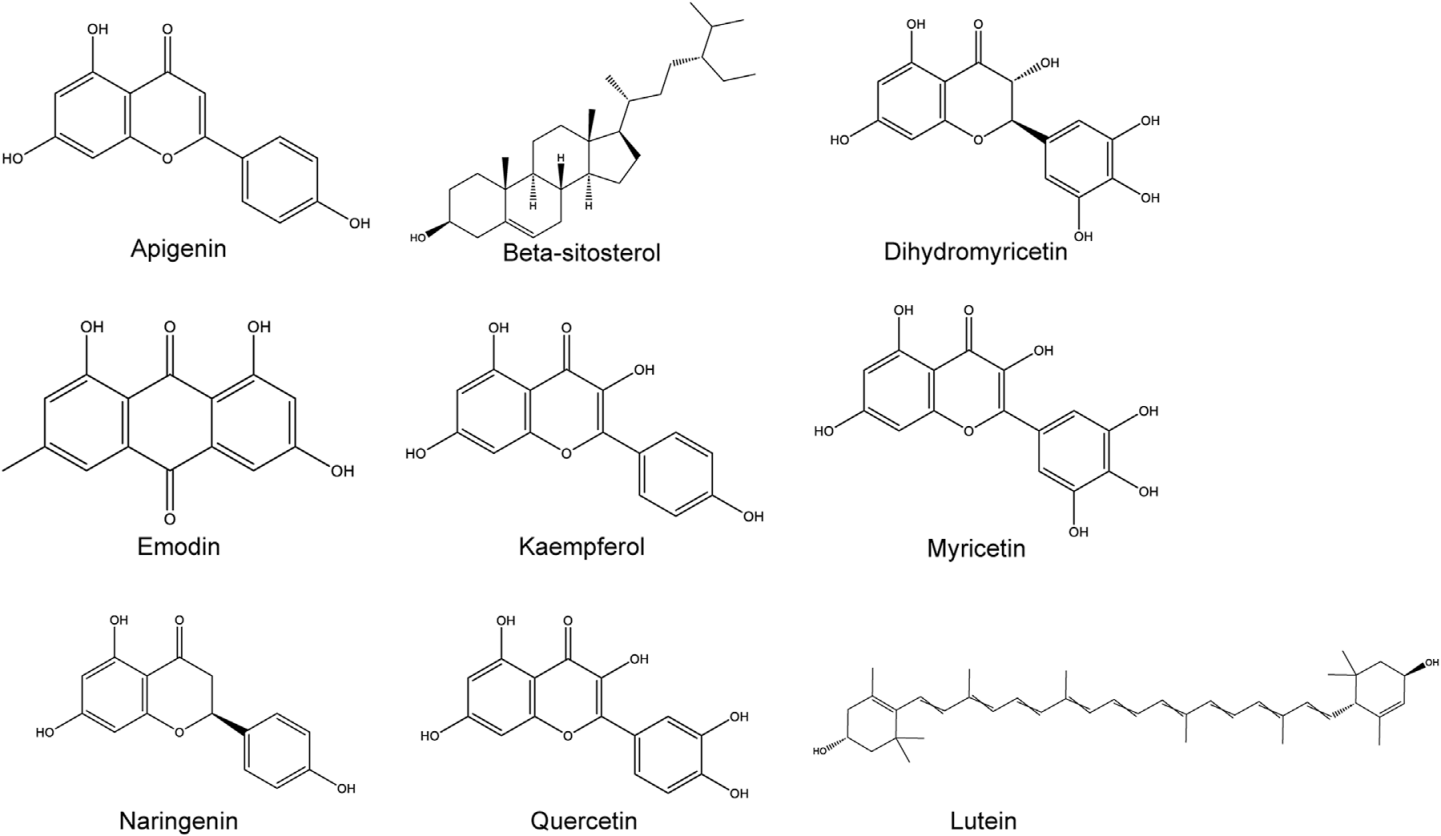
Hovenia dulcis nine chemical components of the chemical structure.

Research on the chemical composition of Hovenia dulcis began in the late 20th century, and a total of 44 species have been documented on the Herb (http://herb.ac.cn/) and TCMSP platforms, including flavonoids, triterpenoid saponins, and alkaloids (Bao-Jun, 2003; Albano, 2008; Li et al., 2021; Sferrazza et al., 2021; Lv et al., 2023). Flavonoids constitute up to 27.61% as the main bioactive components of Hovenia dulcis, (Fu et al., 2023). Of these, nine chemical constituents such as beta-sitosterol, DHM, quercetin naringenin, lutein, myricetin, kaempferol, emodin, and apigenin, were verified to be related to ALD and their chemical results are shown in Figure 1.

## 3 Pathophysiology of ALD

**FIGURE 2:**
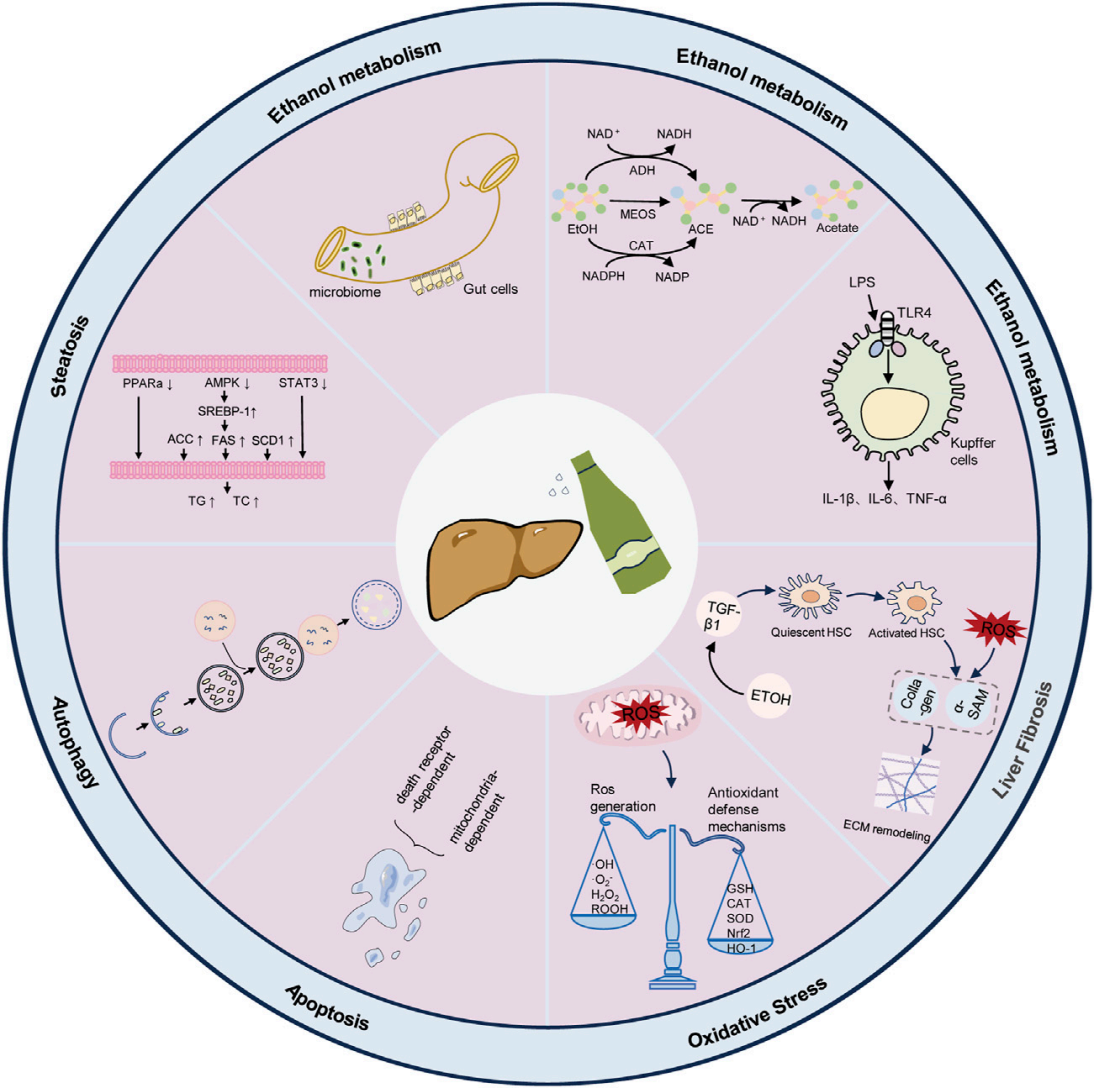
Pathophysiology of alcohol-associated liver disease.

ALD is a chronic liver disease that arises from the excessive consumption of alcohol, typically commencing with alcoholic fatty liver disease. Prolonged alcohol intake disrupts the metabolic capacity of hepatocytes, leading to intracellular lipid accumulation and subsequent progression to more severe manifestations of hepatic injury, such as steatohepatitis, liver fibrosis, cirrhosis, and potentially hepatocellular carcinoma (HCC) (Noh et al., 2011; Peng et al., 2013; Liu S. Y. et al., 2021; Liu Y. S. et al., 2021; Wang et al., 2022). As depicted in Figure 2, the pathophysiology of ALD includes ethanol metabolism and mechanisms in ALD development (Dunn and Shah, 2016; Kong et al., 2019).

### 3.1 Alcohol metabolism

Through the digestive system, alcohol is taken into the bloodstream and affects blood alcohol concentration (BAC), which is mainly processed in hepatocytes (Liu S. Y. et al., 2021). The oxidative conversion of alcohol to acetaldehyde involves three main metabolic processes. The initial step involves the alcohol dehydrogenase (ADH) pathway, facilitated by nicotinamide adenine dinucleotide (NAD) as a cofactor, transforming ethanol into acetaldehyde (Silva et al., 2021). Due to its high reactivity, acetaldehyde can interact with various proteins and form deoxyribonucleic acid (DNA) complexes, thereby influencing protein and genetic material (Shang Zeng and Li, 2022). The second pathway of ethanol metabolism generates reactive oxygen species (ROS) through the microsomal enzyme oxidizing system (MEOS) in the endoplasmic reticulum, leading to various liver reactions, such as oxidative stress and inflammatory damage (Liu S. Y. et al., 2021). The third, more minor pathway is thought to be the catalase (CAT) pathway, which is dependent on nicotinamide adenine dinucleotide phosphate (NADPH) for metabolism (Kong et al., 2019). Subsequently, acetaldehyde is metabolized by the enzyme acetaldehyde dehydrogenase (ALDH) in the mitochondria, leading to the formation of acetate. Acetate is further oxidized to carbon dioxide in various extrahepatic tissues (Chen et al., 2013).

### 3.2 Mechanisms in ALD development

#### 3.2.1 Alcohol metabolism interferes with the liver’s immune response to inflammation

Ethanol triggers unrestrained inflammation and immune turbulence via regulating key cell subsets in the hepatic immune microenvironment. Kupffer cells (KC) (liver-resident macrophages) play a crucial role in the innate immune response against infections and initiating and resolving inflammation (Yao et al., 2017; Chen M. et al., 2023). Chronic alcohol consumption impairs the intestinal mucosal barrier, leading to increase permeability and translocation of intestinal Gram-negative bacteria-derived lipopolysaccharide (LPS) to the liver. As a typical pathogen-associated molecular pattern (PAMP), LPS activates toll-like receptor 4 (TLR4) on macrophages and natural killer cells (NK cells), leading to change macrophage function and increased production of pro-inflammatory cytokines, which results in T cell activation and inflammation (Ling Yang and Lu, 2022; Fang et al., 2023).

#### 3.2.2 Liver sinusoidal endothelial cell dysfunction (LSEC) and liver fibrosis

LSECs are a highly specialized and distinctive micro-vascular cell type that regulates the hepatic micro-environment (Lafoz et al., 2020). LSEC is the first defense barrier, removing and recycling proteins, lipids, and toxins from the blood through highly permeable fenestrae when exposed to blood from the intestinal and systemic circulation (Wu et al., 2023). In addition, they are involved in response to specific injuries by regulating neighboring cells, mainly hepatic stellate cells (HSC).

With alcohol consumption, HSCs and hepatic portal fibroblasts transdifferentiate to myofibroblasts. Activated myofibroblasts produce mainly type I and type III collagen, which cross-links and accumulate in the extracellular matrix (ECM), replacing the injured liver parenchyma and forming a fibrous scar (Bellanti et al., 2023). HSCs can be directly activated by alcohol, acetaldehyde, and ROS or in a paracrine manner by various fibrogenic factors (e.g., transforming growth factor-β, platelet-derived growth factor, and inflammatory cytokines) released from damaged hepatocytes, activated Kupffer cells, and infiltrated immune cells (Ha et al., 2022).

#### 3.2.3 Alcohol-induced mitochondrial dysfunction with activated reactive oxygen species (ROS) storm

Mitochondria are double-membrane organelles involved in various physiological functions within the cell (Park et al., 2022). Typically, mitochondrial abnormalities usually occur in the early stages of alcoholic liver injury that continually produce detrimental effects on hepatocytes. When the liver is chronically exposed to ethanol, mitochondria produce large amounts of reactive nitrogen species (RNS) and ROS, which inactivate components of the respiratory chain by destroying mitochondrial DNA (mtDNA), inducing mitochondrial permeability transition, leading to mitochondrial swelling and rupture, and ultimately hepatocyte death (Su et al., 2023). ROS secreted by mitochondria is scavenged by the cellular antioxidant system. Organisms have developed a variety of antioxidant defense systems to scavenge the accumulation of peroxides in the liver to reduce liver injury caused by oxidative stress (Molina et al., 2003).

#### 3.2.4 Alcohol metabolism-mediated cell death and prosurvival pathways

The complex balance between parenchymal and nonparenchymal cells’ survival and death pathways is critical for regulating ALD progression. In ALD, programmed cell death (PCD), partly caused by ethanol-induced oxidative stress and innate immune responses, is thought to play a central role in the progression of the injury (Miyata and Nagy, 2020). PCD is a spontaneous and programmed cell death mode associated with multiple physiological and pathological processes (Chen Y. et al., 2023). There are four main modes of PCD: apoptosis, necroptosis, pyroptosis, and ferroptosis (Wu et al., 2023). ALD is influenced by dysregulation or hyperactivation of autophagy, which is linked to cellular death and damage.

##### 3.2.4.1 Apoptosis

Apoptosis is a molecular process that plays an essential role in development and tissue homeostasis (Nössing and Ryan, 2023). Apoptosis is a crucial component of various homeostatic processes, including standard cell renewal and normal development and function of the immune system (Miyata and Nagy, 2020). The mechanisms of apoptosis are complex and are now generally recognized as occurring mainly through the death receptor-dependent extrinsic pathway and the mitochondria-dependent intrinsic pathway (Pistritto et al., 2016). The new guidelines distinguish between intrinsic caspase-dependent and intrinsic caspase-independent apoptosis (Guicciardi et al., 2013).

##### 3.2.4.2 Ferroptosis

Ethanol feeding results in iron-dependent ferroptotic cell death (FCD), which is characterized by the accumulation of lipid hydroperoxides to lethal levels in the presence of iron (Wu et al., 2023). Ferroptosis is a ROS-dependent cell death with two major biochemical features: iron accumulation and lipid peroxidation (Tang et al., 2021). Excessive oxidative stress and disruption of the redox system cause lipid peroxidation that further leads to mitochondrial dysfunction, which in turn causes FCD. Thus, the central molecular mechanism of iron death is an imbalance between oxidative damage and antioxidant defenses (Chen Y. et al., 2023).

##### 3.2.4.3 Autophagy

Autophagy is a dynamic process designed to maintain cellular homeostasis by removing lipid droplets, damaged organelles, and faulty proteins from cells to promote cell survival (Dunn and Shah, 2016; Shang Zeng and Li, 2022). There are three main types of autophagy: macro-autophagy, micro-autophagy, and chaperone-mediated autophagy (Glick et al., 2010). Macro-autophagy is the main form of cellular autophagy. It has been documented that acute alcohol exposure induces protective autophagy in the liver, whereas chronic alcohol intake impairs autophagy in hepatocytes (Shang Zeng and Li, 2022). Generally, autophagy is considered an early adaptive response to injury that occurs before apoptosis; however, hyperactivation also results in apoptotic cell death through standard regulators, such as beclin1 and Bcl-2 (Wu et al., 2023).

#### 3.2.5 Disorders of lipid metabolism due to ethanol metabolism

In hepatocytes, lipid uptake, esterification, oxidation, and fatty acid secretion occur. These processes are regulated by hormones, nuclear receptors, and transcription factors to maintain hepatic lipid homeostasis (Fu et al., 2021). Alcohol affects hepatic lipid metabolism through synergistic mechanisms, accumulating fatty acids (FA) and triglycerides (Seitz et al., 2023). Ethanol intake decreases signal transducer and activator of transcription 3 (STAT3), AMP-activated protein kinase (AMPK), and peroxisome proliferative activated receptor alpha (PPARα) levels to inhibit fatty acid oxidation. In addition, alcohol intake upregulates sterol regulatory element binding protein-1 expression (SREBP-1) and lipogenic enzymes. All of these behaviors contribute to dyslipidemia and hepatic fat accumulation (Dunn and Shah, 2016; Zhao et al., 2016; Liu S. Y. et al., 2021).

#### 3.2.6 Alcohol metabolism-mediated intestinal barrier and intestinal flora pathways

Tight junctions (TJ), sticky mucus gel layers, and intestinal cells work together to form the intestinal barrier, which prevents bacteria from passing through (Suzuki, 2020). The intestinal barrier also depends on commensal bacteria, sIgA antibodies, and mucins (Guohua Li and Chen, 2022). In comparison to individuals in good health, patients with alcohol-associated liver disease exhibit a decrease in intestinal bacilli and probiotics (e.g., *Lactobacillus*) and increased *E. coli* and *Enterococcus faecalis* (Dunn and Shah, 2016; Shang Zeng and Li, 2022). Furthermore, studies have demonstrated that augmenting the proportion of intestinal bacilli and firmicutes can serve as a preventive measure against alcoholic liver injury and other chronic liver diseases (Qiu et al., 2019).

## 4 Management of alcohol-associated liver disease

**FIGURE 3:**
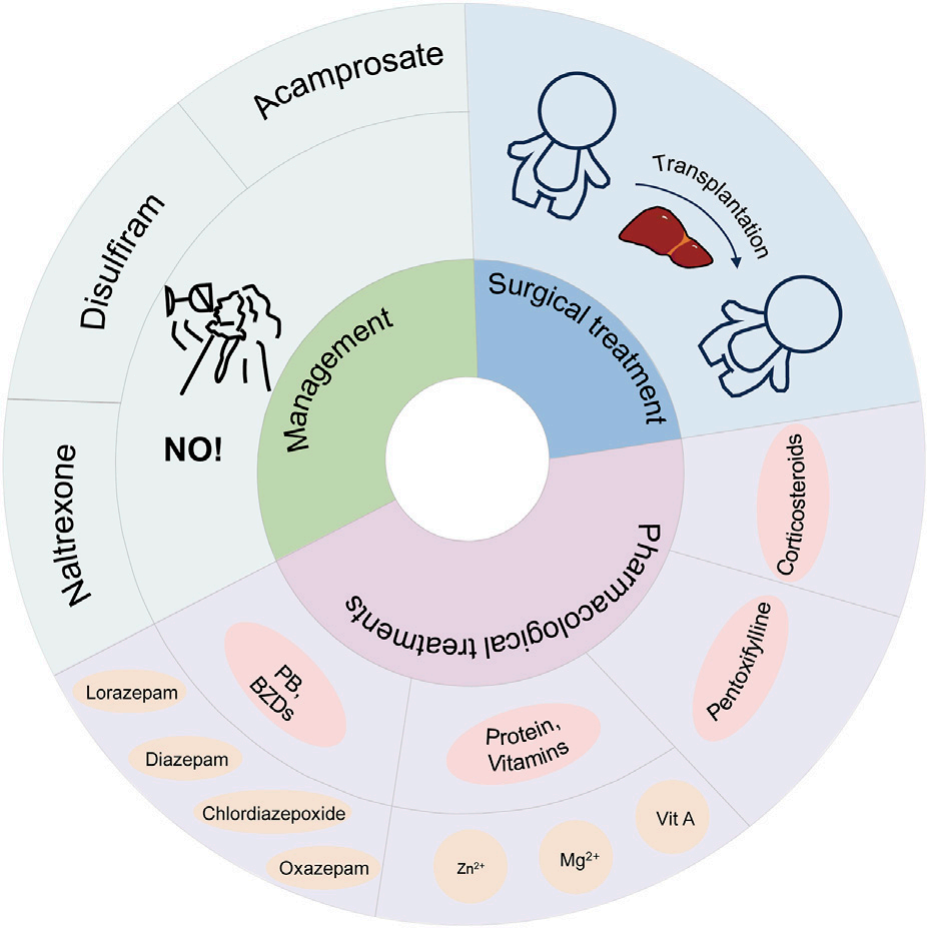
Treatment of alcohol-associated liver disease.

As shown in Figure 3, the treatment of alcohol-associated liver disease consists of management, medication, and surgery.

### 4.1 Management of alcohol-associated liver disease

The most important phase of treatment in patients with alcohol-associated liver disease is achieving alcohol abstinence, since patients who drink alcohol after treatment significantly reduce the efficacy of alcohol-related therapies (Singal et al., 2018). Most patients with alcohol-associated liver disease meet the diagnostic criteria for alcohol-use disorder (AUD) which is a chronic relapsing disease (Erickson et al., 2019). Abuse and dependence are clinical characteristics of alcohol use disorder (AUD), characterized by the involuntary pursuit of alcohol stimulation, a lack of control over alcohol consumption, and depressive symptoms during periods of abstinence (Mason, 2017). Medications for AUD include disulfiram, naltrexone, and acamprosate (Jonas et al., 2014; Hartwell and Kranzler, 2019). Patients with AUD often suffer from concomitant liver damage, but disulfiram and naltrexone can exacerbate liver disease. Therefore, medications that can reduce alcohol intake are minimal (Addolorato et al., 2009). Baclofen is a γ-Aminobutyric acid type B (GABAB) receptor agonist with a deficient hepatic metabolism (10%–15%), and less reported its side effects on liver (Addolorato et al., 2009; Morley et al., 2013).

### 4.2 Treatment of alcohol-associated liver disease

#### 4.2.1 Treatment of alcohol withdrawal syndrome (AWS)

Patients with AUD are often accompanied by Alcohol withdrawal syndrome (AWS). AWS is caused by a dramatic decrease in GABA activity and glutamate overactivity a few hours after cessation of alcohol consumption (Airagnes et al., 2019). Benzodiazepines (BZDs) are the generalized medications used to treat alcoholic AWS, including chlordiazepoxide, oxazepam, diazepam, and lorazepam (Holleck et al., 2019). BZDs act therapeutically by stimulating γ-aminobutyric acid type A (GABA-A) receptors, mimicking their stimulation by alcohol (Airagnes et al., 2019). Phenobarbital (PB) is a standard alternative or adjunct to BZDs and can reduce AWS complications and ICU admissions. PB exerts its effects through the activation of GABA-A receptors and inhibition of N-methyl-D-aspartate (NMDA) receptors (Mo et al., 2016). However, high doses of BZDs induce liver disease and dependence (Singal et al., 2018). PB also has a low margin of safety (Mo et al., 2016). Therefore, there is a need to explore safer medications for use in AWS. Other detrimental conditions may occur during AWS, such as Wernher’s disease, Wernicke-Korsakoff syndrome (WKS) (Airagnes et al., 2019), and alcohol withdrawal delirium (AWD) (Mayo-Smith et al., 2004).

#### 4.2.2 Nutritional therapy

Malnutrition is one of the main adverse consequences of alcohol-associated liver disease, especially in those with severe alcoholic hepatitis. Sufficient protein and vitamins must be administered orally or intravenously as part of nutritional treatment (Griffith and Schenker, 2006). In the case of alcoholism, even if sufficient protein, vitamins, and other nutrients are consumed, the body does not use them effectively because they are not sufficiently absorbed from the intestines into the bloodstream, resulting in malnutrition (Lieber, 2003). Zinc, magnesium, and vitamin A deficiencies are also standard in ALD (McClain et al., 2011).

#### 4.2.3 Corticosteroids

Corticosteroids are used for the treatment of alcoholic hepatitis (AH). One example is prednisolone which is prescribed at a therapeutic dose of 40 mg daily for 4 weeks (Singal et al., 2018). Ramond et al. found that patients treated with prednisolone had a death rate of 12.5%, whereas those given a placebo had a mortality rate of 55% (Ramond et al., 1992). Additionally, prednisolone was found to lower short-term mortality in individuals with severe AH in a clinical investigation (Mathurin et al., 1996). To predict the effect of corticosteroid therapy, the Lille Model was introduced. The Lille Model measures were age, renal insufficiency, albumin, prothrombin time, bilirubin and day 7 bilirubin levels. The model measures from the seventh day of treatment, and patients with scores >0.45 indicate a lack of response to corticosteroids; directing the need for changing the AH treatment to another suitable option (Louvet et al., 2007).

#### 4.2.4 Pentoxifylline

Pentoxifylline is a non-specific phosphodiesterase inhibitor with anti-inflammatory qualities (Tyagi et al., 2011). It is administered at a dose of 400 mg thrice daily to treat severe AH (Akriviadis et al., 2000). Pentoxifylline attenuates the development of hepatic fibrosis by inhibiting TNF-α, which reduces mortality in AH and may decrease the incidence of hepatorenal syndrome (Tyagi et al., 2011). However, in patients with severe AH, there is no additional survival advantage of using pentoxifylline alone compared to corticosteroids (Sidhu et al., 2012). Pentoxifylline is ineffective in patients who are also unresponsive to corticosteroid therapy (Louvet et al., 2008). According to the European Association for the Study of the Liver (EASL), pentoxifylline is a second-line therapeutic agent for patients with severe AH who have contraindications to corticosteroid therapy (Liver, 2012).

### 4.3 Surgical treatment of alcohol-associated liver disease

The Lille Model suggests that patients with severe AH who do not respond to medication have a survival rate of only 25% at 6 months and only 10% at 1 year (Mellinger and Stine, 2020). Liver transplantation (LT) is an option for all patients with end-stage alcohol-associated liver disease (Kong et al., 2019). With the application of antiviral therapy to chronic hepatitis C (HCV), the approach for treating liver disease has shifted to treating ALD (Burra et al., 2010). In 1997, the American Society of Transplantation and the American Association for the Study of Liver Diseases made 6 months of abstinence from alcohol in patients with alcohol-associated liver disease, the minimum criteria for inclusion in alcoholic liver transplantation (Dureja and Lucey, 2010). In the context of liver transplantation, the recurrence rate of alcohol-associated liver disease is 10–50 percent, which is mainly due to cardiovascular disease and neoplastic disease (Iruzubieta et al., 2013). Screening and prevention of these factors in alcoholic patients is therefore essential (Mellinger and Stine, 2020). Brian P Lee et al. showed that patients who received liver transplants early in life had a higher survival rate compared to patients who underwent liver transplants after a strict 6-month abstinence from alcohol (Lee et al., 2017). Late-stage liver transplant patients live only 1.5 years, compared to an average of 6.6 years and even up to 10 years for early-stage liver transplants (Mellinger and Stine, 2020).

## 5 Hovenia dulcis search strategy for the mechanism of action of the treatment of alcohol-associated liver disease

### 5.1 Hovenia dulcis chemical composition retrieval

**FIGURE 4:**
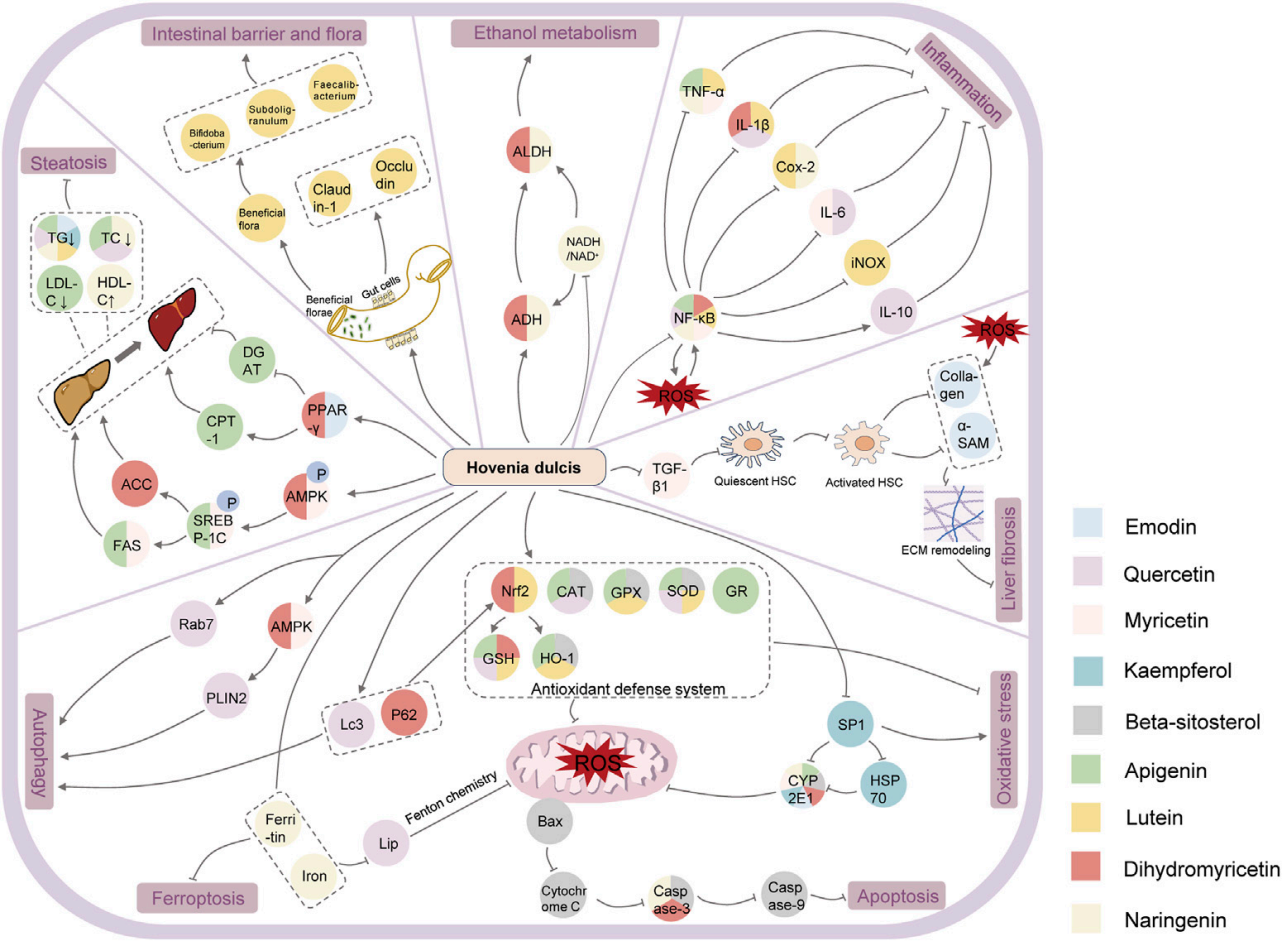
Mechanism of action of Hovenia dulcis chemical constituents in relation to ALD.

In this review, we obtained 44 chemical components of Hovenia dulcis by searching the following databases: TCMSP and Herb, of which the correlation between 9 chemical elements and alcohol-induced liver injury was verified, as shown in Table 1 and Figure 4.

**TABLE 1:** Mechanism of action of Hovenia dulcis chemical constituents in relation to ALD.

No.	Compound	Mechanism							Reported targets	References
		Ethanol metabolism	Inflammatory response	Oxidative stress	Apoptosis	Autophagy	Steatosis	Intestinal barrier		
1	Beta-sitosterol	-	-	+	+	-	-	-	8-OHdG, CYP2E1, MDA, SOD, GPX, CAT, Bax, Caspase-3, Caspase-9	Chen et al. (2020b)
2	Dihydromyricetin/ampelopsin	+	+	+	+	+	+	+	ADH, ALDH, AMPK, Sirt1, Sirt3, PPARγ, PGC-1α, TFAM, ACC, SREBP-1, TNF-α, IL-1β, P62, Keap-1, Nrf2	Shen et al. (2012), Liang and Olsen (2014), Qiu et al. (2017), Zhang et al. (2018), Qiu et al. (2019), Silva et al. (2020), Skotnicová et al. (2020), Chen et al. (2021), Silva et al. (2021), Wang et al. (2022), Janilkarn-Urena et al. (2023)
3	Quercetin	-	+	+	-	+	+	+	Rab7, PLIN2, AMPK, IL-10, IL-1β, LC3Ⅱ, IL-6, TNF-α, ERK, Nrf2, HO-1, Caspase-1, P38, AP-1, NF-κB, GSH, SOD, CAT, P2X7, PI3K, Keap1, Nrf2, Hepc,	Molina et al. (2003), Yao et al. (2007), Vidhya and Indira (2009), Yao et al. (2009), Ambadath et al. (2010), Chen, 2010; Liu et al. (2010), Liu et al. (2012), Tang et al. (2012), Leung and Nieto (2013), Li et al. (2014), Li et al. (2016), Yu et al. (2016), Liu et al. (2018), Zhao et al. (2018), Lee et al. (2019), Zeng et al. (2019), Hou et al. (2020), Pingili et al. (2020), Zhao et al. (2021), Lin et al. (2022), Zhao et al. (2022)
4	Naringenin	+	+	+	+	-	+	+	ADH, ALDH, HDL, TNF-α, IL-6, COX-2, NF-κB, Cyp2e1, ·OH, ROS, RNS, GSH, SOD, CD14	(Seo et al., 2003; Jayaraman and Namasivayam, 2011; Jayaraman et al., 2012; Hernández-Aquino and Muriel, 2018)
5	Lutein	-	+	+	-	-	+	+	TNF-α, IL-1β, NF-κB, COX-2, HO-1, CYP2E1, MDA, SOD, GPX, Nrf-2, Claudin-1, Occludin	(Sindhu et al., 2010; Du et al., 2015; Zhao et al., 2023)
6	Myricetin	-	+	+	-	-	+	+	FAS, SREBP-1c, AMPK, TNF-α, IL-6, TGF-β1, ROS, MDA	(Guo et al., 2019; Ahmad et al., 2022)
7	Kaempferol	-	-	+	-	-	+	-	CYP2E1, ROS, MDA, SP1, ZO-1, Occludin	(Wang et al., 2015; Zhou et al., 2018; Chen et al., 2020a)
8	Emodin	-	-	+	-	-	+	-	PPAR-γ, CYP2E1, α-SAM	Liu et al. (2014)
9	Apigenin	-	+	+	-	-	+	+	SREBP-1c, FAS, LDL-C, NF-κB, TNF-α, GSH, CYP2E1, MDA, SOD, CAT, GSH, PPARα	Wang et al. (2017)

### 5.2 Literature search

**FIGURE 5:**
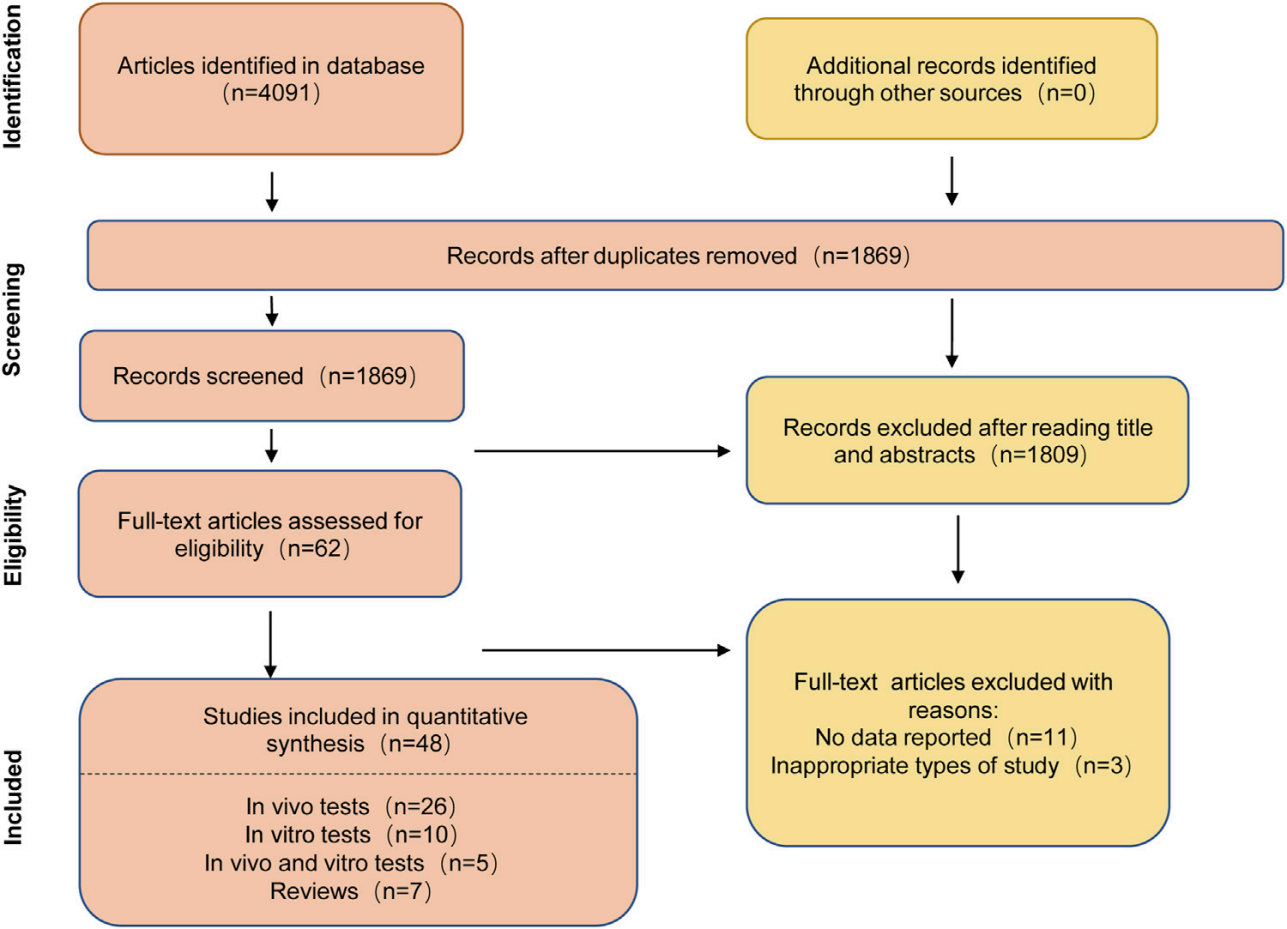
Flowchart for literature search on PubMed.

Based on the above Hovenia dulcis search for each chemical constituent, with “a chemical constituent” and “alcohol-associated liver disease” or “ethanol” or “alcohol” as the keywords, in the electronic databases PubMed, Web of Science (WOS), and the China National Knowledge Infrastructure (CNKI) search. The initial search was conducted on 2 April 2023, and the updated search was conducted on 31 August 2023. The search strategy on PubMed is shown in Supplementary Appendix S1 and the results are shown in Figure 5.

### 5.3 Inclusion exclusion criteria

Inclusion criteria were (a) studies on alcohol-induced liver injury, (b) interventions studied: chemical components of Hovenia dulcis, and (c) studies conducted *in vivo* or *in vitro*. Exclusion criteria were: (a) non-alcohol-induced liver injury, and (b) duplicate studies and titles or abstracts that did not meet the inclusion criteria.

## 6 Mechanism of action of Hovenia dulcis in the treatment of alcohol-associated liver disease

### 6.1 Activation of enzymes related to alcohol metabolism

Ethanol causes a decrease in the activity of ADH and ALDH in the liver, leading to toxic acetaldehyde accumulation. Treatment with DHM and naringenin increases the activity of these two enzymes (Jayaraman and Namasivayam, 2011; Hernández-Aquino and Muriel, 2018; Silva et al., 2020; Chen et al., 2021), accelerating alcohol metabolism and sparing hepatocytes from alcohol-induced effects (Chen et al., 2013). Also, the rate of ethanol metabolism by ADH and the rate of metabolism of acetaldehyde to acetic acid by ALDH is critical for the severity of ethanol damage to the liver (Jayaraman and Namasivayam, 2011). NAD, a coenzyme of dehydrogenase, acts as a cofactor molecule to facilitate the ALD to oxidize ethanol to acetaldehyde (Liu S. Y. et al., 2021). Ethanol intake increases the NADH/NAD ratio (Lingshi Wang, 2001), and naringenin reestablishes the ratio (Hernández-Aquino and Muriel, 2018).

### 6.2 Modulation of inflammatory mediators

#### 6.2.1 LPS/TLR4/NF-κB pathway

Chronic alcohol consumption can damage the intestinal mucosal barrier leading to an increase in its permeability. Lipopolysaccharide (LPS) from intestinal Gram-negative bacteria translocates to the liver, where it binds to Toll-like receptor 4 (TLR4) on kupffer cells, causing its activation and subsequent release of a series of pro-inflammatory factors, such as tumor necrosis factor-alpha (TNF-alpha) and interleukin-1beta (IL-1β), which result in hepatic inflammation and systemic injury (Y, 2014; Shang Zeng and Li, 2022). Nuclear factor κB (NF-κB) is a key transcription factor in regulating inflammatory responses and a key component of intracellular signaling cascades (Xing et al., 2021). Naringenin, Lutein, Myricetin, and Apigenin reduce ethanol-induced levels of the hepatocyte inflammatory factor NF-κB (Jayaraman et al., 2012; Wang et al., 2017; Guo et al., 2019; Zhao et al., 2023). Activation of NF-κB stimulates the production of various inflammatory mediators, such as IL-1β, IL-6, TNF-α, COX-2, and inducible nitric oxide synthase (iNOS) (Jayaraman et al., 2012; Du et al., 2015; Zhao et al., 2016). Among them, IL-1β, IL-6, and TNF-α are synthesized and released upon activation of hepatic stellate cells (Li et al., 2013). These are critical inflammatory factors in alcoholic liver injury and induce further necrosis, apoptosis, and exacerbation of inflammatory response in hepatic cells (Yuan et al., 2022). Quercetin reduces the levels of TNF-α, IL-1β, and IL -6 levels (Chen, 2010); DHM and lutein can reduce TNF-α, and IL-1β (Shen et al., 2012; Zhao et al., 2023), and myricetin can also reduce IL-6, and TNF-α levels (Guo et al., 2019; Ahmad et al., 2022). Naringenin, and apigenin can only reduce the concentration of TNF-α (Jayaraman et al., 2012; Wang et al., 2017). The essential enzyme that starts inflammation, cyclooxygenase-2 (COX-2), is one of NF-κB’s target genes and is expressed by kupffer cells. Normal tissues have low levels of Cox-2 expression; however, an inflammatory stimulation increases Cox-2 expression with subsequent production of prostaglandins (Jayaraman et al., 2012). The inflammatory protective mechanism of naringenin and lutein mainly consists of inhibiting the overexpression of Cox-2 induced by alcohol exposure (Jayaraman et al., 2012; Zhao et al., 2023). Inducible nitric oxide synthase (iNOS) is the enzyme responsible for NO production and is not present in most cells under normal conditions. Its expression is inducible, often associated with inflammation and malignant diseases (Kielbik et al., 2019), and is a downstream gene of NF-κB (Du et al., 2015). Lutein supplementation reverses alcohol-induced changes in iNOS, an indicator of inflammation (Zhao et al., 2023).

#### 6.2.2 Anti-inflammatory factor pathway

Interleukin 10 (IL-10), a robust anti-inflammatory factor, lessens liver damage (Chen, 2010). Quercetin has been shown to positively affect the liver by up-regulating the expression of Heme Oxygenase-1(HO-1) and IL-10, inhibiting the NOD-like receptor thermal protein domain associated protein 3(NLRP3) inflammatory vesicles (Liu et al., 2018).

### 6.3 Inhibition of liver fibrosis

Chronic inflammation in the liver underlies the formation of hepatic fibrosis. Patients with severe ASH who persistently consume alcohol can further progress to fibrosis due to repetitive wound-healing processes in response to chronic inflammation and hepatocellular damage (Ha et al., 2022). TGF-β is the pro-fibrotic cytokine in the liver and contains three isoforms: transforming growth factor-β1 (TGF-β1), TGF-β2, and TGF-β3 (Chang et al., 2023). A crucial inflammatory mediator, TGF-β1 mainly stimulates hepatic stellate cells, kupffer, and perisinusoidal cells—all linked to hepatic fibrosis (Song et al., 2019). Myricetin inhibits the higher TGF-β1 after ethanol exposure, potentially suppressing hepatic fibrosis (Zhao et al., 2018). Alpha-smooth muscle actin (a-SMA) is a marker of activated HSCs, which is responsible for the overproduction of ECM components and the development of fibrosis (Voutilainen et al., 2020). Upon stimulation with TGF-β, HSCs begin to differentiate from a quiescent state into proliferating and fibrotic myofibroblasts that express and secrete α-SMA and type I collagen (CoI-l), CoI-III and CoI-IV, leading to hepatic fibrosis (Chang et al., 2023). Emodin significantly reduces α-SMA and collagen expression in the alcohol-fed mouse group (Liu et al., 2014).

### 6.4 Maintaining mitochondrial stability and reducing oxidative stress damage induced by ethanol intake

Mitochondria are double-membrane organelles involved in a variety of physiological functions within the cell (Park et al., 2022). Silva et al. showed that DHM improves mitochondrial outcomes in the liver of alcohol-fed mice via the AMPK/Sirt-1/PGC-1α signaling axis (Silva et al., 2021).

ROS can originate from mitochondria. Small amounts of free radicals usually maintain redox balance in the human body. However, overproduction of ROS due to excessive alcohol consumption may disturb the balance. Subsequently, oxidative stress may become an essential factor in alcoholic liver injury (Su et al., 2023). Oxidative stress is characterized by an increase in lipid peroxidation associated with alterations in the antioxidant system (Molina et al., 2003). Lipid peroxidation plays a crucial role in hepatic oxidative stress, which was determined by assessing malondialdehyde (MDA) enhancement (Chen, 2010). MDA is the end-product of lipid peroxidation, which can respond to the peroxidation of free radicals *in vivo* and can also indicate the extent to which free radicals indirectly attack cells (Zhao et al., 2010). Beta-sitosterol, DHM, quercetin, lutein, myricetin, kaempferol, and apigenin, can reduce ethanol-induced increases in MDA levels (Chen, 2010; Wang et al., 2015; Wang et al., 2017; Zhang et al., 2018; Guo et al., 2019; Chen Z. et al., 2020; Zhao et al., 2023).

#### 6.4.1 ROS generation pathway

In addition to ADH, the MEOS metabolizes ethanol to acetaldehyde in ALD. The primary components of MEOS are the cytochrome P450 (CYP450) enzymes found in the smooth endoplasmic reticulum (SER), including CYP2E1 and CYP3A, among others (Lu and Cederbaum, 2018). Prolonged alcohol intake leads to the activation of the MEOS system, which can affect NADPH oxidation and ATP consumption, leading to oxidative stress in hepatocytes (Chen et al., 2013). Within the cytochrome P450 family, CYP2E1 is considered the most relevant to ALD due to its high degree of induction and catalytic activity in response to alcohol (Leung and Nieto, 2013). Heat shock protein 70 (HSP70) facilitates the synthesis of CYP2E1 proteins in the endoplasmic reticulum (ER) and their transfer into mitochondria (Zhou et al., 2018). SP1, a ubiquitously expressed transcription factor, transcribes and activates CYP2E1 and HSP70 (Morgan, 1989; Tang et al., 2019). Upon beta-sitosterol, DHM, naringenin, myricetin, kaempferol, emodin, and apigenin treatment, the levels of endogenous CYP2E1 were reduced in liver tissues, which attenuated hepatocyte injury (Jayaraman and Namasivayam, 2011; Liu et al., 2014; Wang et al., 2017; Zhou et al., 2018; Chen Z. et al., 2020; Silva et al., 2020; Ahmad et al., 2022). Kaempferol reduced ethanol-induced protein levels of CYP2E1 in microsomes and mitochondria and decreased the expression of HSP70 and SP1, reducing oxidative stress and increasing cell viability (Zhou et al., 2018).

The P2X7 receptor (P2X7R), is a critical receptor for oxidative stress and inflammation in ALD, induces the generation of activated ROS and imbalance of the antioxidant defense system. P2X7R is an adenosine triphosphate (ATP)-gated ion channel, that can be activated by high levels of ATP to exacerbate oxidative stress through the PI3K/Keap1/Nrf2 pathway. Quercetin treatment eliminates ethanol-induced overexpression of P2X7R and exerts a cytoprotective effect (Zhao et al., 2021).

#### 6.4.2 Antioxidant defense system

The antioxidant defense system can be used to scavenge the accumulation of peroxides in the liver to reduce hepatic injury caused by oxidative stress. The major endogenous antioxidant enzyme systems include superoxide dismutase (SOD), catalase (CAT), glutathione peroxidase (GPX), and glutathione reductase (GR); non-enzymatic endogenous antioxidants are mainly glutathione (GSH) and vitamin E (Molina et al., 2003). Alcohol intake leads to increased levels of MDA and decreased GSH, SOD, and CAT, leading to liver injury. GSH is the first line of defense against free radicals and is the key to antioxidant activity (Vidhya and Indira, 2009; Tang et al., 2012). Treatment with DHM, quercetin, lutein, and apigenin increases the levels of the antioxidant GSH (Donglin Su and Yao, 2009; Vidhya and Indira, 2009; Sindhu et al., 2010; Wang et al., 2017; Hou et al., 2020). In addition, enzymes such as SOD and GPX are essential in scavenging free radicals to prevent their formation (Jayaraman and Namasivayam, 2011). Alcohol use has been linked to a drop in the liver’s MDA and SOD and CAT levels. Treatment with beta-sitosterol, lutein, and apigenin promoted the elevation of SOD and GPX (Sindhu et al., 2010; Wang et al., 2017; Zhao et al., 2018; Chen Z. et al., 2020; Zhao et al., 2023). However, quercetin only promoted the increase of SOD (Vidhya and Indira, 2009). The activity of CAT responds to the cell’s ability to scavenge ROS and its resistance to oxidative damage (Hao and Liu, 2019). The reduction in CAT levels brought on by alcohol can be reversed by beta-sitosterol and apigenin (Wang et al., 2017; Chen Z. et al., 2020). Apigenin treatment can potentially elevate GR levels in alcoholic liver injury (Wang et al., 2017). In the study by Liu et al., the effects of quercetin on GSH, SOD, and CAT levels were guided by pretreatment rather than post-treatment (Liu et al., 2010). Quercetin has also been demonstrated to scavenge oxygen-free radicals more effectively than conventional vitamin C and E forms.

Nrf2 is a protective factor expressed in response to oxidative stress. Excessive ROS disrupt the Keap1-Nrf2 interaction, triggering an effective atomic translocation of Nrf2. The translocated Nrf2 binds to the antioxidant response element (ARE) in blood and enhances the expression of antioxidant and detoxifying enzymes (Lee et al., 2019). Nrf2 has emerged as a potent therapeutic agent for ALD, aiming to protect downstream targets, namely, HO-1 and GSH. HO-1 is the rate-limiting enzyme for heme catabolism. It can degrade heme to ferrous ions, carbon monoxide (CO), and biliverdin, which play a vital role in combating oxidative stress (Liu et al., 2012). Biliverdin then reductase breaks down biliverdin into bilirubin. Heme has a pro-oxidant nature and increases the susceptibility of cells to oxidative stress, while biliverdin and bilirubin exhibit noteworthy anti-inflammatory, antioxidant, and anti-apoptotic properties (Yao et al., 2009). DHM and lutein can activate the antioxidant system by increasing the expression of Nrf2 and its antioxidant product, HO-1 (Zhang et al., 2018; Silva et al., 2020; Chen et al., 2021; Zhao et al., 2023). Quercetin exerts its protective effect against ALD mainly through ERK/Nrf2-mediated upregulation of HO-1 to reduce ROS production (Liu et al., 2012; Zhao et al., 2021). In the mitogen-activated protein kinase (MAPK) signaling pathway, p38 and extracellular regulated protein kinases (ERK) mediated translocation of nuclear factor-erythroid 2-related factor 2(Nrf2) into the nucleus subsequently induces HO-1 activity, with a more critical mediating role for ERK (Yao et al., 2007). It has also been shown that in addition to Nrf2, HO-1 activation is regulated by other transcription factors, including activator protein-1 (AP-1) and nuclear factor (NF-ΚB) (Lee et al., 2019).

### 6.5 Cell death and prosurvival pathways

#### 6.5.1 Inhibition of apoptosis

Chronic alcohol exposure induces multiple forms of cellular stress, including oxidative stress, hypoxia, and endoplasmic reticulum (ER) stress, which activate intrinsic and extrinsic pathways of apoptosis (Miyata and Nagy, 2020). Cell-intrinsic pathways receive tight regulation from the Bcl-2 protein family (Guicciardi et al., 2013). Bax belongs to the Bcl-2 protein family, which plays an essential role in apoptosis (Bruckheimer et al., 1998). Bax appears at the cell membrane in a healthy condition, which gets transferred to the mitochondria in response to apoptotic stressors (DNA damage, misfolded proteins, etc.), increasing the permeability of the cell membrane (Matsuyama et al., 2016). Cytochrome c exists on the inner membrane and then translocates to the cytoplasm (Choi et al., 2022). Bax initiates the intrinsic apoptotic pathway and triggers the release of cytochrome c, which then causes a series of caspase cascade reactions that induce apoptosis (Matsuyama et al., 2016). Beta-sitosterol inhibits alcohol-induced increase in the expression of Bax and inhibits cytochrome c, caspase-3, and caspase-9 elevation (Chen Z. et al., 2020). However, DHM and naringenin only decrease the elevation of caspase-3 after ethanol-mediated (Zhao et al., 2018; Silva et al., 2020).

#### 6.5.2 Inhibition of ferroptosis

The core events of iron toxicity include increased iron accumulation, impaired lipid repair systems, and lipid peroxidation, ultimately leading to membrane destruction and cell death (Wu et al., 2021). Numerous studies have shown that alcohol consumption is associated with iron overload in the liver and decreased hepcidin (Hepc) synthesis, which is thought to be a critical iron mediator of homeostasis (Liu S. Y. et al., 2021). Hepc, a small molecule peptide secreted by the liver, plays a role in iron homeostasis by inhibiting iron uptake and mobilization from tissue stores. Alcohol stimulates iron absorption and hepatic storage by inhibiting the secretion of hepc, which releases free iron from various iron-containing proteins. Free iron in cells exists as a labile iron pool (LIP), i.e., redox-active, weakly chelated iron (Li et al., 2016); that when present in excess catalyzes the Fenton reaction to produce hydroxyl radicals (·OH), exacerbating alcoholic liver injury. Hydroxyl radicals are the most toxic component of ROS (Li et al., 2014; Li et al., 2016). The Fenton reaction occurs within lysosomal compartments, which can accumulate large amounts of redox-active iron and are the primary source of LIP (Li et al., 2016). Quercetin helps to maintain intracellular LIP levels and attenuate intracellular free iron-mediated production of hydroxy radicals (·OH), which can alleviate alcohol-induced liver injury (Li et al., 2014; Li et al., 2016). Ethanol feeding stimulated elevated levels of iron and ferritin in the liver of rats, leading to increased oxidative stress. Whereas naringenin intake significantly reduced the expression of iron and ferritin in the liver (Jayaraman et al., 2012).

#### 6.5.3 Promotion of autophagy

##### 6.5.3.1 Mitophagy

Mitophagy is a selective form of macro-autophagy that mediates mitochondrial degradation in the autolysosomes (Salete-Granado et al., 2023). LC3 is an autophagy-specific protein with three isoforms, LC3A, LC3B, and LC3C, but only LC3-II has been associated with increased phagocytic vesicle membranes. LC3-II is the autophagosomal form of LC3, which is produced by specific protein hydrolysis in the vicinity of the cell and can be a direct response to the degree of active autophagy (Noh et al., 2011). P62 (SQSTM 1) is considered a multifaceted adapter protein. It is thought to be a multidimensional adaptor protein that recognizes ubiquitinated proteins and triggers the autophagic degradation of these proteins via the lysosomal pathway through LC3-II interactions (Qiu et al., 2017; Salete-Granado et al., 2023). Chronic ethanol ingestion leads to impaired autophagy, as evidenced by a reduced LC3. Quercetin restores the levels of LC3. By continuously feeding DHM to C57BL/6 for 6 weeks, Qiu et al., found for the first time that the protective effect of DHM against ethanol-induced liver injury was associated with autophagy induced by the interaction of P62 and the Keap1-Nrf2 system (Qiu et al., 2017). Specifically, DHM can upregulate the expression of P62 and promote ethanol-induced autophagy in mice, activating the positive feedback pathway in the Keap1-Nrf2 pathway, thus exerting its hepatoprotective effect (Qiu et al., 2017; Zhang et al., 2018; Chen et al., 2021).

##### 6.5.3.2 Lipophagy pathway

Lipophagy, which degrades lipid droplets (LDs) mainly through lysosomal acid lipase, is a mode of protection in the body that protects the liver from alcohol-induced steatosis. Specifically, during the lysosomal-centered lipophagy process, LDs in the cytoplasm are engulfed by autophagosomes to be transported to lysosomes, where lipids are broken down by lysosomal acid lipase. Rab7, a critical factor in autophagosome-lysosome fusion, plays an essential role in ALD-related lipophagy. Quercetin intake can restore ethanol-induced impairment of Rab7 cycling to improve lipophagy (Lin et al., 2022). Zeng et al. demonstrated that quercetin reduced perilipin 2 (PLIN2) levels, activated AMPK activity, and increased co-localization of hepatic LC3II and PLIN2 proteins to substantiate the lipophagy-promoting effects of quercetin (Zeng et al., 2019). DHM demonstrated enhanced lipid clearance by way of increased lipophagy activity, shown by the increased interaction and colocalization of p62/SQSTM-1, LC3B, and PLIN-1 proteins (Janilkarn-Urena et al., 2023).

### 6.6 Reduction of steatosis

#### 6.6.1 Lipid metabolism indicators

Hepatic steatosis represents the liver’s earliest and most common response to acute or chronic alcohol exposure (Huo et al., 2018). During steatosis, triglycerides (TG), cholesterol (TC), and phospholipids build up in hepatocytes (Dunn and Shah, 2016). Several studies have shown that quercetin, naringenin, and apigenin reduced alcohol-induced hepatic steatosis. Moreover, these compounds can lower serum TG and TC concentrations, ultimately restoring cell viability (Seo et al., 2003; Zhao et al., 2018; Zhao et al., 2021). Lutein, kaempferol, and emodin only reduced TG levels and had no significant effect on TC (Liu et al., 2014; Wang et al., 2015; Zhao et al., 2023). High-density lipoprotein (HDL-C) is involved in the reverse transport of cholesterol from peripheral tissues and lipids from the vasculature to the liver for clearance (Wierzbicki and Mikhailidis, 2002). Naringin increases the serum HDL-C and HDL-C/total-C ratios, returning these lipid parameters to normal levels and reducing alcohol damage to the liver (Seo et al., 2003). Serum low-density lipoprotein cholesterol (LDL-C) acts oppositely to HDL-C, transporting lipids from the liver into the vasculature and inducing disease. Apigenin reduces the alcohol-induced higher serum values of LDL-C (Wang et al., 2017).

#### 6.6.2 AMPK pathway

AMPK, the classical mammalian upstream serine protein kinase, induces lipolysis and suppresses lipid synthesis. Its activation can lead to a reduction in steatosis and inflammation (Silva et al., 2020; Pang et al., 2021). Ethanol and acetaldehyde inhibit fatty acid oxidation by downregulating the levels of AMPK (Dunn and Shah, 2016; Zhao et al., 2016; Liu S. Y. et al., 2021). DHM and myricetin can decrease AMPK levels (Guo et al., 2019). Increased phosphorylation of AMPK promotes phosphorylation of SREBP-1c (Zhu et al., 2019). One of the critical regulators of adipogenesis, the transcription factor SREBP-1c, plays a role in the transcriptional activation of genes that encode the rate-limiting enzymes in adipogenesis, including acetyl-CoA carboxylase (ACC), fatty acid synthase (FAS), and stearoyl-CoA desaturase 1 (SCD1) (Fang et al., 2019). Myricetin and apigenin significantly inhibited alcohol-induced levels of the liposynthesis gene SREBP-1c (Wang et al., 2017; Guo et al., 2019). FAS catalyzes the condensation of acetyl-CoA and malonyl-CoA to produce long-chain fatty acids in the cytoplasm (Ronnett et al., 2005). Myricetin and apigenin inhibit the elevation of FAS caused by alcohol exposure (Wang et al., 2017; Guo et al., 2019). ACC is the rate-limiting enzyme for the *de novo* synthesis of fatty acids and catalyzes acetyl-CoA conversion to malonyl-CoA (Pang et al., 2021). In the liver, phosphorylation of AMPK inactivates ACC, which decreases fatty acid synthesis and increases fatty acid oxidation (Liu Y. S. et al., 2021). DHM regulates alcohol-induced hepatic steatosis through the AMPK-ACC pathway, which reduces lipid accumulation (Chen et al., 2021).

#### 6.6.3 PPAR pathway

The peroxisome proliferator-activated receptor (PPAR) belongs to the nuclear hormone receptor superfamily and plays a crucial role in glucose and fatty acid metabolism. There are three known members of this superfamily: PPAR-α, PPAR-β/δ, and PPAR-γ. Known as the “energy homeostasis receptor,” PPAR-γ is a crucial regulator in lipid metabolism. PPAR-γ regulates several downstream genes involved in fatty acid oxidation, such as carnitine palmitoyltransferase-1 (CPT-1) (Zheng and Cai, 2019). DHM and emodin decreased PPAR-γ caused by ethanol exposure (Liang and Olsen, 2014; Liu et al., 2014; Chen et al., 2021). Fatty acid catabolism occurs in mitochondria and is regulated by CPT-1, the rate-limiting step in entering fatty acids into mitochondria (Ronnett et al., 2005). Diacylglycerol acyltransferase (DGAT), a microsomal enzyme widely expressed in mammalian tissues, is also a key enzyme in triglyceride synthesis (Chen and Farese, 2000). Pre-administration of apigenin decreased hepatic DGAT protein expression in mice with alcohol-induced liver injury, while hepatic CPT-1 protein expression was inversely increased. These findings show that pretreatment with apigenin reduced hepatic lipid synthesis and increased fatty acid utilization, indicating that apigenin may be able to avoid abnormalities of hepatic lipid metabolism (Wang et al., 2017).

### 6.7 Protection of the intestinal barrier and intestinal flora

The intestinal barrier system depends on the interactions between several barrier components, including the dense mucus gel layer, immunoglobulin A, commensal bacteria, and intercellular TJs (Suzuki, 2020; Guohua Li and Chen, 2022). TJs play a pivotal role in establishing the physical barrier among these components. The transmembrane proteins claudin and occludin, as well as the intracellular plaque proteins zonula occludens (ZO) and neuregulin, make up the TJs structure (Suzuki, 2020). TJs such as claudin-1 regulate epithelial and endothelial cells by forming TJ chains, paracellular barrier integrity, and paracellular barrier permeability between epithelial cells and endothelial cells (Zwanziger et al., 2015). Occludin, the first transmembrane protein of the epithelial cell tight junction to be identified, plays an essential role in regulating tight junction integrity (Rao, 2009). Alcohol-induced reductions in claudin-1 and occludin can be reversed by supplementation with lutein (Zhao et al., 2023). Short-chain fatty acids (SCFAs) produced by gut flora are the major contributing factors to the development of the TJ. SCFAs are part of the intestinal biological barrier produced in the colon. The concentration of SCFA in the colon’s lumen is approximately 60% acetate, 25% propionate, and 15% butyrate. The short-chain fatty acid butyrate improves mucosal inflammation and oxidative status, strengthens the epithelial defense barrier, and modulates visceral sensitivity and intestinal motility (Canani et al., 2011). Kaempferol restores TJ protein ZO-1 and occludin, as well as the levels of butyrate receptor GPR109A and butyrate transporter SLC58A proteins in ethanol-exposed mice (Chen J. et al., 2020).

Chronic alcohol intake leads to bacterial overgrowth and dysbiosis in the small and large intestines of animals and humans (Fukui, 2015). Beneficial intestinal flora like Bifidobacterium and Faecalibacterium contribute to the concentration of butyrate in the digestive system, which reduces intestinal inflammation and preserves intestinal integrity (Ralli et al., 2021). Lutein reduces the amount of damage caused by alcohol while increasing the amount of Bifidobacterium, Subdoligranulum, and Faecalibacterium (Zhao et al., 2023).

## 7 Conclusion and perspectives

Worldwide, the prevalence of alcohol-associated liver disease is progressively rising. In the early stages, patients can regain their liver function by abstaining from alcohol and practicing exercise. However, as the disease progresses, drug intervention or even liver transplantation is required. Currently, the primary drugs used to treat ALD are disulfiram, BZDs, corticosteroids, and pentoxifylline. However, these drugs have a certain degree of hepatotoxicity and dependence. Therefore, there is still a lack of adequate and safe drugs in this field. Herbal medicine can provide a potential solution for controlling and treating ALD due to its multi-pathway and multi-target characteristics.

Numerous studies have shown that Hovenia dulcis contains a wide spectrum of pharmacological activities that can be used to treat a variety of liver diseases. Therefore, exploring its pharmacological mechanism of action is beneficial to provide a new direction for the treatment of alcohol-associated liver disease. This review, based on the current research on Hovenia dulcis and its derived natural compounds, retrieved nine chemical components related to ALD, which are beta-sitosterol, DHM, quercetin, naringenin, lutein, myricetin, kaempferol, emodin, apigenin. The potential protective mechanisms of these nine chemicals against ALD involve ethanol metabolism, immune response, hepatic fibrosis, oxidative stress, autophagy, lipid metabolism, intestinal barrier, and many more. The antioxidant mechanism is involved in every chemical component, lipid metabolism is involved in eight components, and the inflammatory response is involved in six components, so it can be deduced that Hovenia dulcis plays a significant role in the treatment of ALD by detoxifying and lowering lipids, as well as anti-inflammatory and hepatoprotective effects.

Despite this study of the therapeutic effects of Hovenia dulcis' chemical composition on ALD, the current experimental study still has some limitations. (1) Currently, herbs with Hovenia dulcis as the main ingredient are primarily used in the research and development of healthcare products, failing to develop into therapeutic drugs. In the same time, the research on the pharmacological effects of using Hovenia dulcis to treat ALD is mainly *in vivo* and *in vitro* experiments, with a lack of double-blind, multi-center, and randomized controlled clinical trials. Hence, its safety, especially for long-term use, remains to be determined. (2) The material basis of Hovenia dulcis‘s efficacy in treating ALD is closely related to its unique content of natural compounds. Therefore, it is crucial to clarify the basis of Hovenia dulcis‘s pharmacological effects and quality standards through the International Organisation for Standardisation. To overcome these limitations, we should conduct more analyses to find further mechanisms and targets for Hovenia dulcis treatment of alcohol-associated liver disease. (1) Establish a high-quality Hovenia dulcis clinical research protocol to facilitate large-scale, multi-center, controlled trials to assess the efficacy and safety of Hovenia dulcis in treating alcohol-associated liver disease to promote the development and clinical application of the drug. (2) Improve the Hovenia dulcis quality recognition standards to reflect its true quality more accurately. In the same time, exploring the interactions of the various active ingredients in Hovenia dulcis and their compatibility with other natural substances for drugs is also an important direction for future research.

## 8 Scope statement

We would like to submit the enclosed manuscript entitled “Hovenia dulcis: A Chinese medicine that plays an essential role in alcohol-associated liver disease” for your consideration as a review in “Frontiers in Pharmacology”. In this manuscript, we reviewed the mechanism of action of Hovenia dulcis in the treatment of alcohol-associated liver disease and its targets**,** providing us with new ideas for the treatment of alcohol-associated liver disease. All authors of this research manuscript were directly involved in the planning, implementation, and/or analysis of this study. The authors declare no competing interests. The contents of this manuscript are not copyrighted and have not been previously published. We deeply appreciate your consideration of our manuscript, and we look forward to receiving comments from the reviewers.

## Figures and Tables

**Table:** 

**ACC**	acetyl-CoA carboxylase
**ADH**	alcohol dehydrogenase
**AH**	alcoholic hepatitis
**ALD**	alcohol-associated liver disease
**ALDH**	acetaldehyde dehydrogenase
**AMPK**	AMP-activated protein kinase
**AP-1**	activator protein-1
**ARE**	antioxidant response element
**α-SMA**	alpha-smooth muscle actin
**ATP**	adenosine triphosphate
**AUD**	alcohol-use disorder
**AWD**	alcohol withdrawal delirium
**AWS**	alcohol withdrawal syndrome
**BAC**	blood alcohol concentration
**BZDs**	benzodiazepines
**CAT**	catalase
**CNKI**	the China National Knowledge Infrastructure
**CO**	carbon monoxide
**CPT-1**	carnitine palmitoyltransferase-1
**CYP2E1**	cytochrome P450 2E1
**CYP450**	cytochrome P450
**DGAT**	diacylglycerol acyltransferase
**DHM**	dihydromyricetin
**DNA**	deoxyribonucleic acid
**EASL**	the European Association for the Study of the Liver
**ECM**	extracellular matrix
**ER**	endoplasmic reticulum
**ERK**	extracellular regulated protein kinases
**FAS**	fatty acid synthase
**GABA**	gamma aminobutyric acid
**GABA-A**	gamma aminobutyric acid type A
**GABAB**	gamma aminobutyric acid type B
**GPX**	glutathione peroxidase
**GR**	glutathione reductase
**GSH**	glutathione
**HCC**	hepatocellular carcinoma
**HCV**	hepatitis C
**HDL-C**	high-density lipoprotein
**Hepc**	ferromodulin
**HO-1**	heme oxygenase-1
**HSCs**	hepatic stellate cells
**HSP70**	heat shock protein 70
**IL-1β**	interleukin-1β
**IL-10**	interleukin 10
**iNOS**	inducible nitric oxide synthase
**LC3**	microtubule-associated protein 1 light chain 3
**LDL-C**	low-density lipoprotein cholesterol
**LDs**	lipid droplets
**LIP**	labile iron pool
**LPS**	lipopolysaccharide
**LT**	liver transplantation
**MAPK**	mitogen-activated protein kinase
**MDA**	malondialdehyde
**MEOS**	microsomal enzyme oxidizing system
**NAD**	nicotinamide adenine dinucleotide
**NADPH**	nicotinamide adenine dinucleotide phosphate
**NK cells**	natural killer cells
**NLRP3**	the NOD-like receptor thermal protein domain associated protein 3
**NMDA**	N-methyl-D-aspartate
**NF-κB**	nuclear factor kappa B
**Nrf2**	nuclear erythroid 2-related factor 2
**P2X7R**	the P2X7 receptor
**PB**	phenobarbital
**PLIN2**	perilipin 2
**PPAR**	peroxisome proliferator-activated receptor
**PPARα**	peroxisome proliferative activated receptorα
**ROS**	reactive oxygen species
**ROS/RNS**	reactive oxygen/reactive nitrogen species
**SCD1**	stearoyl-CoA desaturase 1
**SCFAs**	short chain fatty acid
**SOD**	superoxide dismutase
**SER**	smooth endoplasmic reticulum
**SQSTM1/P62**	sequestosome 1
**SREBP-1**	sterol regulatory element binding protein-1 expression
**STAT3**	signal transducer and activator of transcription 3
**TC**	cholesterol
**TG**	triglycerides
**TGF-β1**	transforming growth factor-β1
**TJ**	tight junctions
**TNF-α**	tumor necrosis factor-alpha
**WHO**	the world health organization
**WKS**	Wernicke-Korsakoff syndrome
**WOS**	web of science
**Hovenia dulcis**	Hovenia dulcis Thunb. (Rhamnaceae)
**ZO**	zonula occludens
**8-OHdG**	8-hydroxy-2-deoxyguanosine
**OH**	hydroxyl radicals
