# Supplementary material for: Hovenia dulcis: a Chinese medicine that plays an essential role in alcohol-associated liver disease
Source: Front Pharmacol. 2024 Apr 8;15:1337633. doi: 10.3389/fphar.2024.1337633 (PMC11033337; doi:10.3389/fphar.2024.1337633)
Supplement: Supplementary file 1 [file Table1.pdf]

Supplementary Material

Hovenia dulcis: A Chinese medicine that plays an essential role in alcohol-associated liver disease

Yi-Xiang He[2, 3]†, Meng-Nan Liu[3]†, Yang-Yang Wang[2, 3]†, Hao Wu[3], Mei Wei[2, 3], Jin-Yi Xue[3], Yuan Zou[3], Hui Chen[1, 2, 3*], Xin Zhou[1, 2, 3*], Zhi Li[1, 2, 3*]

*** Correspondence:** Corresponding Author: email@uni.edu

Zhi Li:lizhi-swmu@126.com

Xin Zhou:zhouxinmm@swmu.edu.cn

Hui Chen: fairysusan@126.com

Appendix: The approach for searching PubMed

Search Strategy in PubMed

Search	Query	Items found
#1	21302-79-4 [Title/Abstract] AND # Alcoholic Liver Disease [Title/Abstract]	0
#2	21302-79-4 [Title/Abstract] AND # Alcohol [Title/Abstract]	0
#3	21302-79-4 [Title/Abstract] AND # Ethanol [Title/Abstract]	0
#4	(2r,3r)-aromadendrin [Title/Abstract] AND # Alcoholic Liver Disease [Title/Abstract]	0
#5	(2r,3r)-aromadendrin [Title/Abstract] AND # Alcohol [Title/Abstract]	0

#6	(2r,3r)-aromadendrin [Title/Abstract] AND # Ethanol [Title/Abstract]	0
#7	3,4,5-trihydroxybenzoic acid [Title/Abstract] AND # Alcoholic Liver Disease [Title/Abstract]	0
#8	3,4,5-trihydroxybenzoic acid [Title/Abstract] AND # Alcohol [Title/Abstract]	4
#9	3,4,5-trihydroxybenzoic acid [Title/Abstract] AND # Ethanol [Title/Abstract]	4
#10	(+)-3,5,7,3',5'-pentahydroxyflavanone [Title/Abstract] AND # Alcoholic Liver Disease [Title/Abstract]	0
#11	(+)-3,5,7,3',5'-pentahydroxyflavanone [Title/Abstract] AND # Alcohol [Title/Abstract]	0
#12	(+)-3,5,7,3',5'-pentahydroxyflavanone [Title/Abstract] AND # Ethanol [Title/Abstract]	0
#13	Ampelopsin [Title/Abstract] AND # Alcoholic Liver Disease [Title/Abstract]	0
#14	Ampelopsin [Title/Abstract] AND # Alcohol [Title/Abstract]	6
#15	Ampelopsin [Title/Abstract] AND # Ethanol [Title/Abstract]	12
#16	Ampeloptin [Title/Abstract] AND # Alcoholic Liver Disease [Title/Abstract]	0
#17	Ampeloptin [Title/Abstract] AND # Alcohol [Title/Abstract]	6
#18	Ampeloptin [Title/Abstract] AND # Ethanol [Title/Abstract]	12
#19	Apigenin [Title/Abstract] AND # Alcoholic Liver Disease [Title/Abstract]	3

#20	Apigenin [Title/Abstract] AND # Alcohol [Title/Abstract]	70
#21	Apigenin [Title/Abstract] AND # Ethanol [Title/Abstract]	260
#22	Arachic acid [Title/Abstract] AND # Alcoholic Liver Disease [Title/Abstract]	0
#23	Arachic acid [Title/Abstract] AND # Alcohol [Title/Abstract]	7
#24	Arachic acid [Title/Abstract] AND # Ethanol [Title/Abstract]	8
#25	Aromadedrin [Title/Abstract] AND # Alcoholic Liver Disease [Title/Abstract]	0
#26	Aromadedrin [Title/Abstract] AND # Alcohol [Title/Abstract]	0
#27	Aromadedrin [Title/Abstract] AND # Ethanol [Title/Abstract]	0
#28	Aromadendrin [Title/Abstract] AND # Alcoholic Liver Disease [Title/Abstract]	0
#29	Aromadendrin [Title/Abstract] AND # Alcohol [Title/Abstract]	2
#30	Aromadendrin [Title/Abstract] AND # Ethanol [Title/Abstract]	19
#31	Beta-sitosterol [Title/Abstract] AND # Alcoholic Liver Disease [Title/Abstract]	1
#32	Beta-sitosterol [Title/Abstract] AND # Alcohol [Title/Abstract]	111
#33	Beta-sitosterol [Title/Abstract] AND # Ethanol [Title/Abstract]	240

#34	Emodin [Title/Abstract] AND # Alcoholic Liver Disease [Title/Abstract]	0
#35	Emodin [Title/Abstract] AND # Alcohol [Title/Abstract]	30
#36	Emodin [Title/Abstract] AND # Ethanol [Title/Abstract]	92
#37	G6657_SIGMA [Title/Abstract] AND # Alcoholic Liver Disease [Title/Abstract]	0
#38	G6657_SIGMA [Title/Abstract] AND # Alcohol [Title/Abstract]	0
#39	G6657_SIGMA [Title/Abstract] AND # Ethanol [Title/Abstract]	0
#40	Gallocatechin [Title/Abstract] AND # Alcoholic Liver Disease [Title/Abstract]	0
#41	Gallocatechin [Title/Abstract] AND # Alcohol [Title/Abstract]	12
#42	Gallocatechin [Title/Abstract] AND # Ethanol [Title/Abstract]	26
#43	Hovenicacid [Title/Abstract] AND # Alcoholic Liver Disease [Title/Abstract]	3
#44	Hovenicacid [Title/Abstract] AND # Alcohol [Title/Abstract]	10
#45	Hovenicacid [Title/Abstract] AND # Ethanol [Title/Abstract]	6
#46	Hovenine A [Title/Abstract] AND # Alcoholic Liver Disease [Title/Abstract]	0
#47	Hovenine A [Title/Abstract] AND # Alcohol [Title/Abstract]	0

#48	Hovenine A [Title/Abstract] AND # Ethanol [Title/Abstract]	0
#49	Jujubogenin [Title/Abstract] AND # Alcoholic Liver Disease [Title/Abstract]	0
#50	Jujubogenin [Title/Abstract] AND # Alcohol [Title/Abstract]	1
#51	Jujubogenin [Title/Abstract] AND # Ethanol [Title/Abstract]	2
#52	Kaempferol [Title/Abstract] AND # Alcoholic Liver Disease [Title/Abstract]	7
#53	Kaempferol [Title/Abstract] AND # Alcohol [Title/Abstract]	97
#54	Kaempferol [Title/Abstract] AND # Ethanol [Title/Abstract]	457
#55	Lutein [Title/Abstract] AND # Alcoholic Liver Disease [Title/Abstract]	2
#56	Lutein [Title/Abstract] AND # Alcohol [Title/Abstract]	139
#57	Lutein [Title/Abstract] AND # Ethanol [Title/Abstract]	105
#58	Mmethyl behenate [Title/Abstract] AND # Alcoholic Liver Disease [Title/Abstract]	0
#59	Methyl behenate [Title/Abstract] AND # Alcohol [Title/Abstract]	0
#60	Methyl behenate [Title/Abstract] AND # Ethanol [Title/Abstract]	1
#61	Methyl caprate [Title/Abstract] AND # Alcoholic Liver Disease [Title/Abstract]	0
#62	Methyl caprate [Title/Abstract] AND # Alcohol [Title/Abstract]	3

#63	Methyl caprate [Title/Abstract] AND # Ethanol [Title/Abstract]	1
#64	Methyl henicosanoate [Title/Abstract] AND # Alcoholic Liver Disease [Title/Abstract]	0
#65	Methyl henicosanoate [Title/Abstract] AND # Alcohol [Title/Abstract]	0
#66	Methyl henicosanoate [Title/Abstract] AND # Ethanol [Title/Abstract]	0
#67	Methyl heptoate [Title/Abstract] AND # Alcoholic Liver Disease [Title/Abstract]	0
#68	Methyl heptoate [Title/Abstract] AND # Alcohol [Title/Abstract]	1
#69	Methyl heptoate [Title/Abstract] AND # Ethanol [Title/Abstract]	0
#70	Methyl hexoate [Title/Abstract] AND # Alcoholic Liver Disease [Title/Abstract]	0
#71	Methyl hexoate [Title/Abstract] AND # Alcohol [Title/Abstract]	9
#72	Methyl hexoate [Title/Abstract] AND # Ethanol [Title/Abstract]	4
#73	Methyl icosanoate [Title/Abstract] AND # Alcoholic Liver Disease [Title/Abstract]	0
#74	Methyl icosanoate [Title/Abstract] AND # Alcohol [Title/Abstract]	0
#75	Methyl icosanoate [Title/Abstract] AND # Ethanol [Title/Abstract]	0
#76	Methyl margarate [Title/Abstract] AND # Alcoholic Liver Disease [Title/Abstract]	0

#77	Methyl margarate [Title/Abstract] AND # Alcohol [Title/Abstract]	0
#78	Methyl margarate [Title/Abstract] AND # Ethanol [Title/Abstract]	0
#79	Methyl myristate [Title/Abstract] AND # Alcoholic Liver Disease [Title/Abstract]	0
#80	Methyl myristate [Title/Abstract] AND # Alcohol [Title/Abstract]	5
#81	Methyl myristate [Title/Abstract] AND # Ethanol [Title/Abstract]	1
#82	Methyl nonadecanoate [Title/Abstract] AND # Alcoholic Liver Disease [Title/Abstract]	0
#83	Methyl nonadecanoate [Title/Abstract] AND # Alcohol [Title/Abstract]	1
#84	Methyl nonadecanoate [Title/Abstract] AND # Ethanol [Title/Abstract]	1
#85	Methyl nonylate [Title/Abstract] AND # Alcoholic Liver Disease [Title/Abstract]	0
#86	Methyl nonylate [Title/Abstract] AND # Alcohol [Title/Abstract]	0
#87	Methyl nonylate [Title/Abstract] AND # Ethanol [Title/Abstract]	0
#88	Methyl octylate [Title/Abstract] AND # Alcoholic Liver Disease [Title/Abstract]	0
#89	Methyl octylate [Title/Abstract] AND # Alcohol [Title/Abstract]	33
#90	Methyl octylate [Title/Abstract] AND # Ethanol [Title/Abstract]	17

#91	Methyl pentadecanoate [Title/Abstract] AND # Alcoholic Liver Disease [Title/Abstract]	0
#92	Methyl pentadecanoate [Title/Abstract] AND # Alcohol [Title/Abstract]	0
#93	Methyl pentadecanoate [Title/Abstract] AND # Ethanol [Title/Abstract]	0
#94	Methyl valerate [Title/Abstract] AND # Alcoholic Liver Disease [Title/Abstract]	0
#95	Methyl valerate [Title/Abstract] AND # Alcohol [Title/Abstract]	5
#96	Methyl valerate [Title/Abstract] AND # Ethanol [Title/Abstract]	0
#97	mono-Methyl suberate [Title/Abstract] AND # Alcoholic Liver Disease [Title/Abstract]	0
#98	mono-Methyl suberate [Title/Abstract] AND # Alcohol [Title/Abstract]	0
#99	mono-Methyl suberate [Title/Abstract] AND # Ethanol [Title/Abstract]	0
#100	Myricetin [Title/Abstract] AND # Alcoholic Liver Disease [Title/Abstract]	2
#101	Myricetin [Title/Abstract] AND # Alcohol [Title/Abstract]	23
#102	Myricetin [Title/Abstract] AND # Ethanol [Title/Abstract]	141
#103	Naringenin [Title/Abstract] AND # Alcoholic Liver Disease [Title/Abstract]	3
#104	Naringenin [Title/Abstract] AND # Alcohol [Title/Abstract]	52

#105	Naringenin [Title/Abstract] AND # Ethanol [Title/Abstract]	110
#106	Norharman [Title/Abstract] AND # Alcoholic Liver Disease [Title/Abstract]	0
#107	Norharman [Title/Abstract] AND # Alcohol [Title/Abstract]	18
#108	Norharman [Title/Abstract] AND # Ethanol [Title/Abstract]	10
#109	Octacosanol [Title/Abstract] AND # Alcoholic Liver Disease [Title/Abstract]	0
#110	Octacosanol [Title/Abstract] AND # Alcohol [Title/Abstract]	27
#111	Octacosanol [Title/Abstract] AND # Ethanol [Title/Abstract]	6
#112	Pentahydroxybufostane [Title/Abstract] AND # Alcoholic Liver Disease [Title/Abstract]	0
#113	Pentahydroxybufostane [Title/Abstract] AND # Alcohol [Title/Abstract]	0
#114	Pentahydroxybufostane [Title/Abstract] AND # Ethanol [Title/Abstract]	0
#115	Perlolyrine [Title/Abstract] AND # Alcoholic Liver Disease [Title/Abstract]	0
#116	Perlolyrine [Title/Abstract] AND # Alcohol [Title/Abstract]	3
#117	Perlolyrine [Title/Abstract] AND # Ethanol [Title/Abstract]	1
#118	Quercetin [Title/Abstract] AND # Alcoholic Liver Disease [Title/Abstract]	23

#119	Quercetin [Title/Abstract] AND # Alcohol [Title/Abstract]	298
#120	Quercetin [Title/Abstract] AND # Ethanol [Title/Abstract]	1241
#121	Sitogluside [Title/Abstract] AND # Alcoholic Liver Disease [Title/Abstract]	0
#122	Sitogluside [Title/Abstract] AND # Alcohol [Title/Abstract]	0
#123	Sitogluside [Title/Abstract] AND # Ethanol [Title/Abstract]	1
#124	Stigmasterol [Title/Abstract] AND # Alcoholic Liver Disease [Title/Abstract]	1
#125	Stigmasterol [Title/Abstract] AND # Alcohol [Title/Abstract]	54
#126	Stigmasterol [Title/Abstract] AND # Ethanol [Title/Abstract]	106
#127	Trochol [Title/Abstract] AND # Alcoholic Liver Disease [Title/Abstract]	0
#128	Trochol [Title/Abstract] AND # Alcohol [Title/Abstract]	0
#129	Trochol [Title/Abstract] AND #3	0
#130	Vanillic acid [Title/Abstract] AND # Alcoholic Liver Disease [Title/Abstract]	0
#131	Vanillic acid [Title/Abstract] AND # Alcohol [Title/Abstract]	133
#132	Vanillic acid [Title/Abstract] AND #3	121
